# Migrating Huns and modified heads: Eigenshape analysis comparing intentionally modified crania from Hungary and Georgia in the Migration Period of Europe

**DOI:** 10.1371/journal.pone.0171064

**Published:** 2017-02-02

**Authors:** Peter Mayall, Varsha Pilbrow, Liana Bitadze

**Affiliations:** 1 The University of Melbourne, Parkville, Victoria, Australia; 2 Ivane Javakhishvili Institute of History and Ethnology, Tbilisi, Georgia; Medical University of South Carolina, UNITED STATES

## Abstract

An intentionally modified head is a visually distinctive sign of group identity. In the Migration Period of Europe (4^th^– 7^th^ century AD) the practice of intentional cranial modification was common among several nomadic groups, but was strongly associated with the Huns from the Carpathian Basin in Hungary, where modified crania are abundant in archaeological sites. The frequency of modified crania increased substantially in the Mtskheta region of Georgia in this time period, but there are no records that Huns settled here. We compare the Migration Period modified skulls from Georgia with those from Hungary to test the hypothesis that the Huns were responsible for cranial modification in Georgia. We use extended eigenshape analysis to quantify cranial outlines, enabling a discriminant analysis to assess group separation and identify morphological differences. Twenty-one intentionally modified skulls from Georgia are compared with sixteen from Hungary, using nineteen unmodified crania from a modern population as a comparative baseline. Results indicate that modified crania can be differentiated from modern unmodified crania with 100% accuracy. The Hungarian and Georgian crania show some overlap in shape, but can be classified with 81% accuracy. Shape gradations along the main eigenvectors indicate that the Hungarian crania show little variation in cranial shape, in accordance with a two-bandage binding technique, whereas the Georgian crania had a wider range of variation, fitting with a diversity of binding styles. As modification style is a strong signifier of social identity, our results indicate weak Hunnic influence on cranial modification in Georgia and are equivocal about the presence of Huns in Georgia. We suggest instead that other nomadic groups such as Alans and Sarmatians living in this region were responsible for modified crania in Georgia.

## Introduction

Intentional cranial modification is a process whereby the head of an infant is purposefully moulded by applying external pressure to achieve a desired shape. Parents or carers start the process as soon as an infant is born and continue it for the first three or four years of life while the cranial bones are still malleable. Once the bones have ossified the cranial vault assumes the intended shape and it is irreversibly modified. As a cultural practice intentional cranial modification was a worldwide phenomenon [1–3]. With independent occurrences spanning over ten millennia the motives for modification may have varied.

Whatever the motives, intentional cranial modification falls within the realm of body modifications along with tattoos, body piercings, and dental engravings that serve to denote affiliation with particular social or ideological groups. In as much as cranial modification is permanent and irreversible, yet not self-initiated, and in that it affects the most visible part of the body, the modified head serves as a strong physical signifier of conferred social identity and provides an excellent medium for studying human agency in promoting social identity [4, 5]. The social significance of cranial modification has been researched most intensively in South America and Mesoamerica, where cranial modification was ubiquitous for several thousand years [6–9]. The incidence and style of modification sheds light on social status, migration patterns, boundaries between social groups and changing social identity [6,7,10,11].

The purpose of this paper is to examine the social significance of intentional cranial modification in the Mtskheta region of the Republic of Georgia, where there was a dramatic increase in the practice of modification during the Migration Period paralleling that seen among Eurasian nomads under the influence of Huns. Roughly coinciding with the collapse of the Western Roman Empire, the Migration Period from the 4^th^ century AD to the 7^th^ century AD saw a large-scale movement of Germanic and Eurasian nomads into and around Roman territory [12]. The Huns based in Hungary are attributed with triggering the Migration movement, destabilising the Roman Empire and influencing the increased uptake of cranial modification in Europe [12–16]. Comparing modified skull shape in Georgia and Hungary will allow us to determine the presence of Huns in Georgia and understand their social influence.

**Fig 1:**
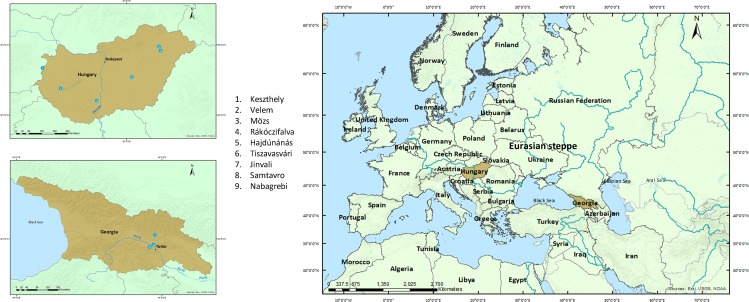
Map of Europe highlighting Hungary and Georgia and the locations of sites with modified crania.

The practice of cranial modification has been documented in Europe sporadically since the sixth millennium BC [2], well before the arrival of the Huns. Indo-Iranian nomadic-pastoralists such as the Scythians, Sarmatians, and Alans who lived in the region of the Eurasian Pontic steppes and the Caucasus had practised cranial modification since the fourth millennium BC [17–20]. The Scythians dominated the steppe region of the northern Black Sea (Fig 1) from the 7^th^ - 3^rd^ centuries BC until they were overtaken by the Sarmatians from the northern Caucasus [21]. By the 2nd century AD the Alans had overthrown the Sarmatians and occupied the territory from the Aral Sea to the Caspian Sea extending to the north of the Black Sea including the northern Caucasus (Fig 1). In the late third century AD the Huns displaced the Alans, who dispersed throughout Western Europe and North Africa, eventually settling in the northern Caucasus, in present-day Ossetia [22]. Several modified crania from Central Asia, the Trans-Ural region, and the Carpathian basin, dated prior to the arrival of the Huns, have been attributed to these groups [23–27].

The Huns were a heterogeneous band of nomadic warriors who had their origins in China and Inner Mongolia [17,28,29]. They arrived in Europe via Inner Asia incorporating several Central Asian Turkic and Scytho-Sarmatian groups within their ranks [17,28,30]. They had modified heads by the time they arrived in Europe, yet skeletal remains of the Chinese and Mongolian predecessors of the Huns show no signs of cranial modification [14,17,18,31,32]. It is believed that they picked up the practice in their journey towards Europe [2,14,18,31]. They settled for the most part in the Carpathian basin in Hungary, which has been described as the epicentre of Hunnic influence [18]. Their presence in Europe was short-lived, from about 370 AD to 460 AD, but they had an immense and lasting influence on the history and polity of the region. The incursions of the Huns on the Alans and Goths north of the Black Sea triggered the Migration Period and they were largely responsible for destabilising the Western Roman Empire [12,16,18,30,33]. Their exploits, exaggerated to mythical proportions by the historians of the day, are strong indication that they wielded considerable regional power [12,18,28,29].

After the arrival of Huns, the incidence of modified crania rose sharply in burials in the Carpathian basin in Hungary, to around 50 to 80 per cent of crania [2,15,23,34–37]. At the same time modified crania start to appear in increased frequency in nomadic burials throughout Europe [2,15,23,37,38]. If we postulate that the Huns were influential and promoted the renewed practice of cranial modification in Europe [12–18, 30, 32,33], we can use cranial modification to examine the physical presence and social sphere of influence of Huns. One such area is the Republic of Georgia where modified crania have been noted sporadically since the Bronze Age. During the Migration Period, starting from about 4^th^ century AD, however, modified skulls are far more frequently encountered in the region of Mtskheta, the capital of Iberian kingdom of Eastern Georgia, although there are no records that the Huns settled in Georgia during this period.

There are records that the Huns travelled across the Caucasus into Armenia through the Derbend Pass in 395 AD and returned to the Hungarian plain in the same way [17,18]. There are suggestions that Georgian noblemen sought the help of Huns to quell internal uprisings [18], but there are no firm records of Huns settling in Georgia. There are, however, ample references to the Alans and Sarmatians in Georgia and in the region of Mtskheta, as allies in strengthening the reign of Iberian rulers or rebels in carrying out raids on the Iberian kingdom [39,40]. It is known that the Huns had a close symbiotic relationship with the Alans and Sarmatians throughout the region of the Caucasus and Rome [22,38,40], subjugating them and including them within their ranks [16,19,21,29]. The question then is: can the modified heads in Georgia be attributed to Huns, or did they belong to another contemporaneous group practising cranial modification? In this paper we compare the pattern of cranial modification in the Migration Period in Georgia with that in Hungary, to determine the likelihood and extent of Hunnic influence in Georgia. If the Georgian modified crania are Hunnic in origin, then they should be indistinguishable from Hungarian modified crania. Alternatively, if they were more diverse in origin they will only loosely resemble Hungarian Hunnic modified crania.

## Materials and methods

Previous studies assessing patterns of cranial modification have variously used visual approaches [41,42], linear measurements [43], angles between landmarks [44], arc and chord measurements [45], and Elliptical Fourier Analysis [46], to study the shape of modified and unmodified crania. These approaches have progressively allowed for a more objective characterisation of modified crania, validating the ability of the naked eye in initially differentiating modified from unmodified crania. Our aim in this study is firstly to quantitatively separate modified crania from unmodified crania and secondly to differentiate modified crania from Georgia and Hungary. All the modified crania in our study were of the annular or circumferential type produced by the application of a circular bandage around the head, although there are variations, where a second vertical bandage is applied to the circular one. The annular form of modification is most commonly encountered in West Asia, Europe, the Caucasus, Trans-Ural and Siberian regions [2,13,15,23,47–50]. As with Perez [9], we use a geometric morphometric approach of Procrustes superimposition to remove differences related to orientation, translation and scale, so as to highlight differences in the annular modification style. We do not expect cranial shape to be ethnically or geographically relevant because the Georgian and Hungarian populations in the Migration Period were ethnically diverse, and overall cranial shape is a poor indicator of geographic origin [51]. Unlike Perez [9], we use extended eigenshape analysis [52–54], because this method allows us to divide the cranial outline into segments and study the difference between similarly modified Georgian and Hungarian crania more closely.

### Sample

Our study is based on 21 modified crania from Georgia, 16 modified crania from Hungary and 19 unmodified crania from The University of Melbourne (Table 1). The Georgian crania came from the permanent osteological repositories of the Georgia National Museum in Mtskheta and the Ivane Javakhishvili Institute of History and Ethnology, University of Tbilisi. The Hungarian crania were studied at the permanent repository of the Hungarian Natural History Museum, Budapest. The sample localities are shown in Fig 1. We used dates provided by the museum or the original excavators to restrict our study to Migration Period sites (Table 1). The crania from Samtavro in Georgia and Keszthely-Fenékpuszta in Hungary have associated radiocarbon dates (Table 1). The unmodified crania from The University of Melbourne are part of a teaching collection of mixed worldwide provenance acquired after 1850 [55]. They were selected precisely because they have no history of intentional modification and permitted us an unbiased shape comparison with modified crania. All specimens are accessible in the specified repositories and teaching collection. The museums and university provided permission by electronic communication to undertake non-invasive scanning (no permit numbers provided).

**Table 1:** Materials used in the study.

	Sites	Specimen Numbers	Dates	Males	Females	Total
Georgia						
	Samtavro	1. 1593 2. 1601 3. 1594 4. 1596 5. 1618 6. 1614 7. 1619 8. 1620 9. 1621 10. 1635 11. 1638 12. 1645 13. 1654 14. 1871 15. 1964 16. 1880	4–6 c AD [56]	3	13	
	Jinvali	1. 2383 2. 2379 3. 2402 4. 2020	4–6 c AD^1^	1	3	
	Nabagrebi	1. 1711	6–7 c AD^1^	1		
Total				5	16	21
Hungary						
	Hajdúnánás-Furjhalom	1. 151229 2. 162250 3. 190280 4. 273396 5. 290415 6. 337493 7. 355515 8. 8221072 9. 8221073	3–5 c AD^2^	4	5	
	Keszthely-Fenékpuszta	1. 2009264 2. 20092612	4–7 c AD [57]		2	
	Mohacs	1. 5258	5–6 c AD^2^		1	
	Mözs	1. 11893	5 c AD [58,59]	1		
	Rakoczifalva	1. 200711 2. 200717	5–6 c AD [60]	1	1	
	Velem-Szentvid	1. 5111	5 c AD^2^		1	
Total				6	10	16
Melbourne	Worldwide	1. 516200174 2. 516200364 3. 516200369 4. 516200370 5. 516200371 6. 516200377 7. 516200379 8. 516200381 9. 516200383 10. 516200387 11. 516200396 12. 516200419 13. 516200391 14. 516200400 15. 516200406 16. 516200416 17. 516200429 18. 516200434 19. 516200438	AD 1850–1980 [55]	9	10	19

^1^ Georgia National Museum.

^2^ Hungarian Natural History Museum.

In Hungary the modified cranial collection includes infants, juveniles, sub-adults and adults, with an equitable distribution of sexes. In Georgia 80% of the modified crania are adult female. There are no juveniles or sub-adults in the Migration Period sample in Georgia. To ensure an equal comparison we used only adult individuals in our study. We assessed adulthood through the eruption of the maxillary third molars and fusion of the cranial sutures. Sex was determined based on cranial characters such as prominence of glabella, sharpness of supraorbital margin, size of mastoid process, robusticity of nuchal crest and strength of mental eminence [61]. Because our study focused on the intact cranial vault, we were forced to exclude individuals with incomplete crania. Thus, our sample sizes and locality representations are reduced, and the sex breakdown deviated from the available sample, such that the Georgian sample included 76% females and the Hungarian sample 63% females. We did not focus on sex differences in our analyses, but pooled the sexes because Procrustes superimposition removes differences of scale.

### Data acquisition

We used a Next Engine laser scanner to obtain three-dimensional (3D) images of the crania. It uses multiple laser stripes to image the object. In the wide view, which was used to image crania in this study, it has a resolution of 0.038 cm with 60 points per cm.

**Fig 2:**
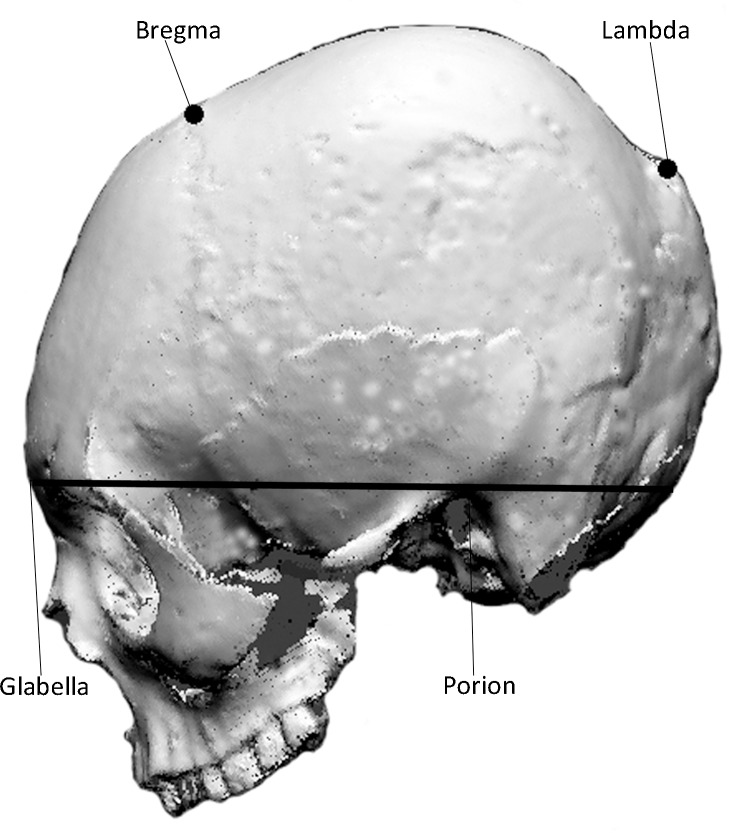
Cranial outline showing bregma and lambda and straight line demarcating cranial base.

To acquire data for digitisation we marked the landmarks, bregma and lambda, on the 3D scans and positioned the scans in left lateral view such that a horizontal line extended from glabella to porion to the occipital region. We then converted the 3D scans into two dimensional (2D) images (Fig 2). We used the 2D outline of the cranial vault for analysis. Frieß and Baylac [46] have previously demonstrated that annular or circumferential modification results in shape changes to the face and the basioccipital region. However, in the absence of the face and cranial base, 2D outlines of the cranial vault preserve sufficient shape variation to characterise such modification. The cranial vault was the most suitable for the ancient crania in our study as in half of the available specimens the cranial base was missing or incomplete. We confined our study to crania in which the vault was intact. The horizontal line connecting glabella to porion and extending to the occipital region was used to demarcate the inferior margin of the cranium. Using the TPSDig program [62] we placed landmarks commencing at the occiput, continuing anti-clockwise to lambda, bregma and glabella, and obtained XY coordinates for these. We quantified the cranial outline with 200 points and acquired XY coordinates for these points (S1 File).

The advantage of using extended eigenshape analysis is that it allowed us to focus on parts of cranial outline that showed the greatest changes in curvature while excluding the inferior straight segment from glabella to occiput from the outline analysis as it contributed no curvature. The landmarks and corresponding segments were selected so as to split the cranial outline into three curved segments and study differences in binding styles in each of the homologous segments: occiput to lambda (occipital), lambda to bregma (parietal), and bregma to glabella (frontal). In the case of four skulls from Samtavro where the base was deficient and porion was absent, we reconstructed this region using a base from a complete skull with the same glabella to occipital length. We placed a minimum of three corresponding points on the skull with the deficient base and the base of the substitute skull. We then used the align function of the Next Engine scanner software to align and unite the skulls. This allowed us to increase our sample size and take a complete outline for the tpsDig program, although we later excluded the base in the extended eigenshape analysis.

We conducted intraclass correlation studies [63,64] to determine the error rate in identifying landmarks on the cranial outline. Both intraobserver and interobserver error studies revealed high interclass correlations of 0.99 to 1.00 between two sets of measurements taken on a random sample of five specimens each from Georgia, Hungary and Melbourne. Having repeatedly identifiable landmarks and outlines ensured that each segment represented topologically homologous curves.

### Eigenshape analysis

Extended Eigenshape analysis implements the techniques described in Krieger and MacLeod [65] and MacLeod [52–54] through the internet-accessible Extended Eigenshape Morpho-tool [66]. Although each specimen was marked with 200 points around the outline, segments with a more complicated structure were assigned a greater number of coordinate points (semi-landmarks). The maximum numbers of points encountered in individual segments were then interpolated to homologous segments across all specimens. Thus, individual segments across all specimens had the same number of equidistant points as the specimen with the most complex curvature. This ensured that fine details of shape change were captured and the outlines from each specimen had the same number of semi- landmarks. This procedure produced a single curve in the form of XY coordinates for each specimen. Procrustes superimposition using a Generalized Least Squares procedure helped to minimize the discrepancies in landmark configurations related to location and orientation and to resize each outline to unit centroid size to scale size differences. The XY coordinates were then converted to phi-functions [67], which are cumulative changes in the angle between tangents to the curve from point to point, expressed in radians. The phi functions effectively remove size, position and rotation from the outline data and align the data. A pairwise matrix of covariances between the phi functions was used to perform a singular value decomposition (similar to a principal component analysis), which re-configured and reduced the shape variation in the sample to a series of hierarchical eigenvectors or eigenshapes. The scores (eigenscores) of specimens on the eigenshapes were used to plot and visualize shape changes along the morphospace [66]. To further explore the discrimination between Georgian, Hungarian and modern crania we used the eigenshapes as variables in a discriminant analysis procedure. We used a step-wise procedure for the selection of eigenshapes, so that only those eigenshapes that were highly significant in discriminating groups were selected in an iterative manner to be included in the analysis (p<0.01 for F-value of variable to be entered, p< 0.10 for F-value of a variable to be removed). In each analysis no more than six eigenshapes contributed to maximum separation. The significance of F-values (p < 0.05) for the pairwise Mahalanobis distances helped determine the statistical difference between groups.

In the second stage of the analysis we studied the contribution of each of the landmarked segments in differentiating between the Georgian and Hungarian modified crania. The open curve procedure of extended eigenshape analysis allowed us to plot the eigenvectors showing shape changes, and a discriminant analysis helped to study the contribution of each segment in differentiating the two groups. In circumferential modification, where a single or a double binding results in significant shape differences in the regions of the frontal, parietal and occipital, separate analyses allowed us to study the shape differences in greater detail.

## Results

### Analysis of cranial outline

**Fig 3:**
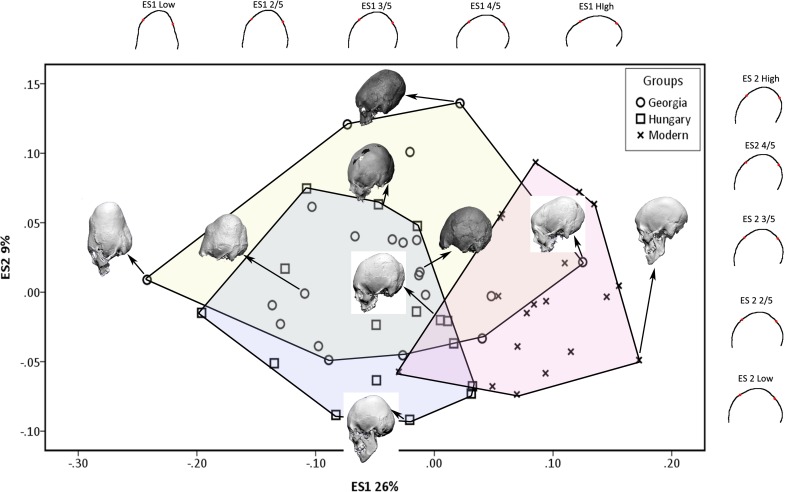
Eigenshape (ES) 1 and 2 showing scatter of cranial outlines with convex hulls demarcating each group, shape changes along the first and second morphospaces, and example crania demonstrating the variance.

**Fig 4:**
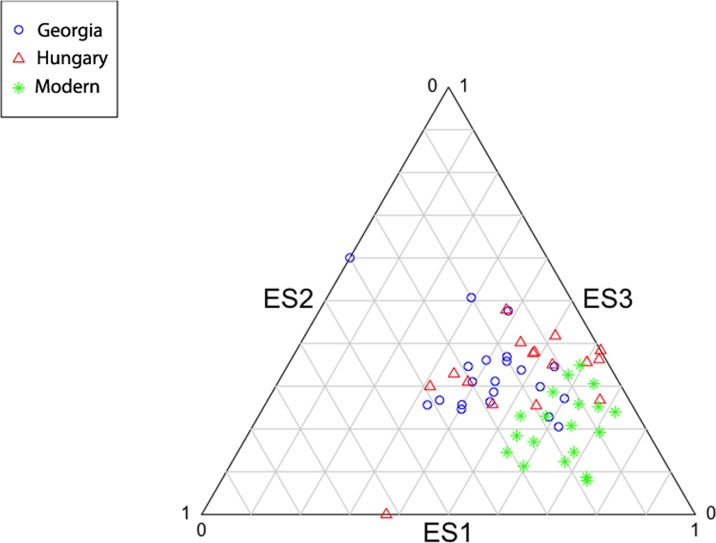
Ternary plot of the first three eigenshapes showing clustering pattern of crania.

The first two eigenshapes, ES1 and ES2, account for respectively, 26% and 9% of the variance in cranial shape. Each subsequent eigenshape captured a smaller proportion of variance, indicating substantial variation in modified cranial shape, forming a continuum with unmodified cranial shape. Fig 3 is a scatterplot of the cranial outlines on the first two eigenshapes with convex hulls demarcating each group. Also shown are the shape changes along the first two morphospaces and example crania demonstrating the variation in cranial shape. The example crania are oriented in the manner used for analysis. As the cranial outlines and example crania show, the first eigenshape plots the change from severely modified, tall and antero-posteriorly narrow cranial vaults to unmodified, shallow, antero-posteriorly wider vaults. The separation of the unmodified crania from the modified crania is seen along this axis. The second eigenshape separates, to some extent, the Georgian crania from the Hungarian ones. It plots the shape change from a mildly modified cranial vault with curved frontal, parietal and occipital outlines to an oblique modification with supero-posteriorly oriented frontal and parietal, but rounded occipital outline. With the first two eigenshapes covering merely 35% of the variance in shape, the clusters in the scatterplot are not distinct. A ternary diagram (Fig 4) of the first three eigenshapes covering 43% of variance shows clear separation of the unmodified modern crania from the modified crania. High eigenscores on ES1 and ES2, with low eigenscores on ES3 cause the samples to cluster at the lower right corner, with some degree of separation of the Georgian and Hungarian crania along ES2 and ES3.

**Fig 5:**
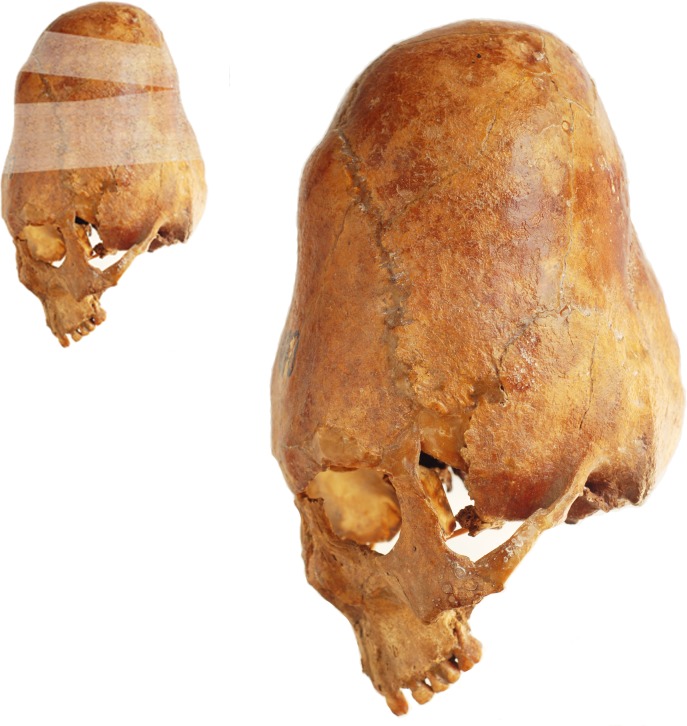
Georgian cranium used in this study demonstrating the effect of the annular form of modification, with several circular bandages resulting in a tall, vertical cranial profile. The orientation of the cranium is that used in the eigenshape analysis in this study.

**Fig 6:**
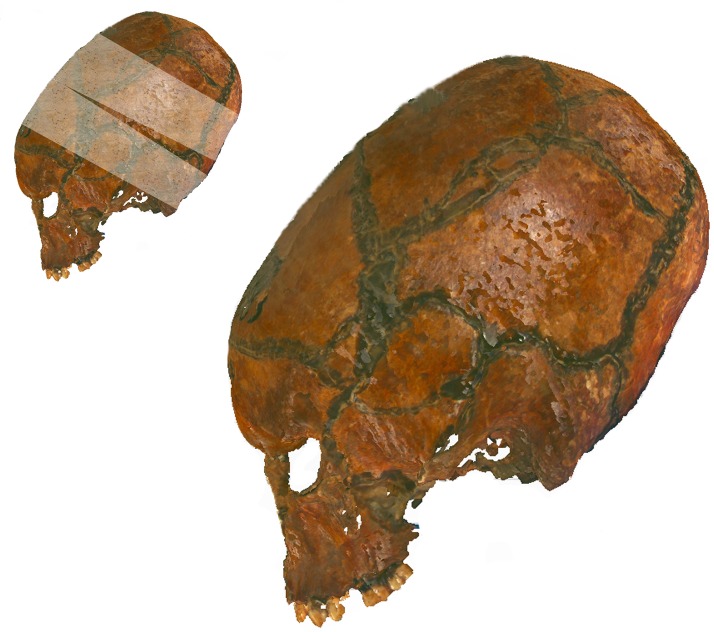
Georgian cranium used in this study demonstrating the annular form of modification, with several circular bandages resulting in an oblique cranial profile. The orientation of the cranium is that used in the eigenshape analysis in this study.

**Fig 7:**
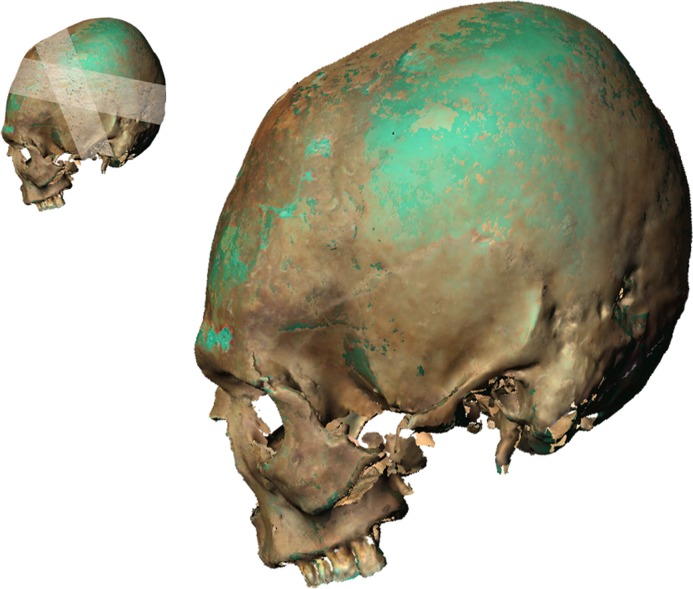
Hungarian cranium used in this study demonstrating the two bandage technique of annular modification. In addition to the circular bandages, a vertical bandage in the region of the bregma restricts cranial height and results in a bregmatic depression. The orientation of the cranium is that used in the eigenshape analysis in this study.

The Georgian crania display varying forms of modification from mild to extreme (Figs 5–7). In the extreme form the skull base is narrow and there is a compensatory bulge superiorly. The frontal, squamous temporal, parietal and occipital bones are elongated and the superior parts of the parietal and occipital bones extend postero-superiorly. This is consistent with the use of a circular bandage secured in a ring-like manner from frontal to occipital. By positioning the bandages either high or low on the frontal and occipital and altering the tightness of the bandages the cranium is made to look tall, with vertical frontal and occipital profiles and bulging parietal profile (Fig 5), or low, with obliquely sloping frontal, occipital and parietal profiles (Fig 6). A milder form of modification is also seen, which restricts the height of the cranial vault and results in a bregmatic or postbregmatic depression along the vertical path of the bandage. This is consistent with adding vertical bandages to the circular ones, going from the crown of the head to the mandible (Fig 7). Our study indicates that both techniques of modification, along with variation in the first technique, were used in Georgia.

The Hungarian crania, by comparison, are constrained in their modification and do not display the tall, erect, oblique or shallow cranial vaults. This would suggest that they were modified using the second technique of two bandages, with one concentrically fitted bandage going from forehead to the nape of the neck across the temporal region, and another bandage going from the crown of the head to continue under the chin (Fig 7).

**Fig 8:**
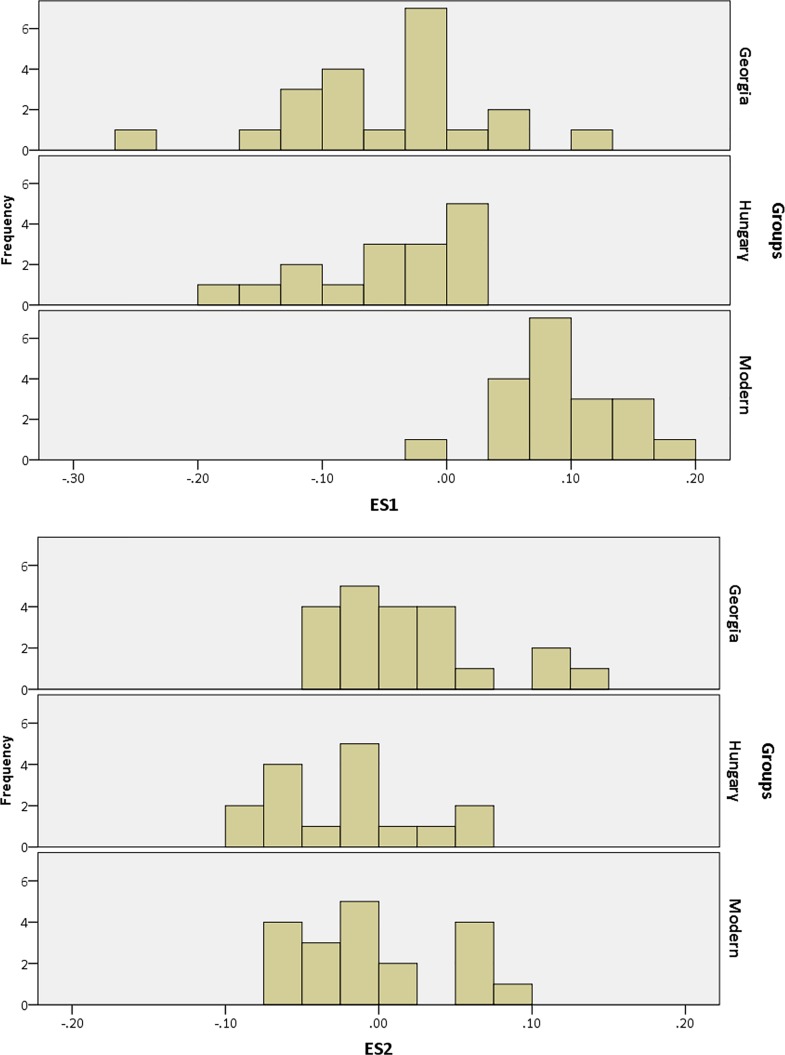
Histograms depicting the eigenscores of Georgian, Hungarian and modern crania on ES1 and ES2.

**Fig 9:**
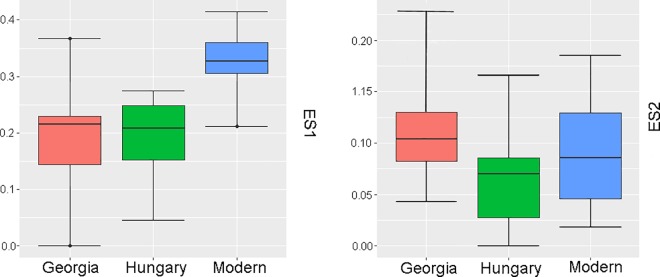
Box-and-whisker plots showing eigenshape scores for the Georgian, Hungarian and modern crania on ES1 and ES2.

The Georgian crania have a wider scatter in all plots compared with the Hungarian and modern crania. Increased variation in cranial form in Georgia also becomes clear by examining the histograms and box-and-whisker plots showing the values for each group along the first two eigenshapes (Figs 8 and 9). The Georgian crania have greater variance in cranial shape, while the Hungarian crania, although distinct from the modern unmodified crania, are restricted in shape.

The step-wise discriminant function analysis used six eigenshapes to maximize discrimination; the pair-wise distinctions were statistically significant. Table 2 shows the accuracy of classification in the step-wise and cross-validation approaches. In the step-wise procedure the unmodified modern crania were discriminated with 100% accuracy. The Hungarian and modern crania were reciprocally 100% distinct; one Georgian cranium with a long and low cranial vault fell into the modern group (Fig 3). The Hungarian and Georgian crania, discriminated with 81% accuracy, had some overlap. The cross-validation procedure maintained the distinction of the Hungarian crania from the unmodified group, although one unmodified specimen fell into each of the Hungarian and Georgian groups. The Georgian crania had greater overlap with the unmodified modern sample in cross-validation, with three specimens classified as modern. The Hungarian and Georgian groups also showed overlap and reduced cross-validated classification accuracy.

**Table 2:** Classification accuracy from discriminant analysis of cranial outline.

Groups	Georgia (N)	Hungary (N)	Modern (N)	Percentage accuracy
**Georgia**	17	3	1	81.0%
**Hungary**	3	13	0	81.3%
**Modern**	0	0	19	100%
**Cross-validation**				
**Georgia**	11	7	3	52.4%
**Hungary**	5	11	0	68.8%
**Modern**	1	1	17	89.5%

### Analysis of cranial segments

**Fig 10:**
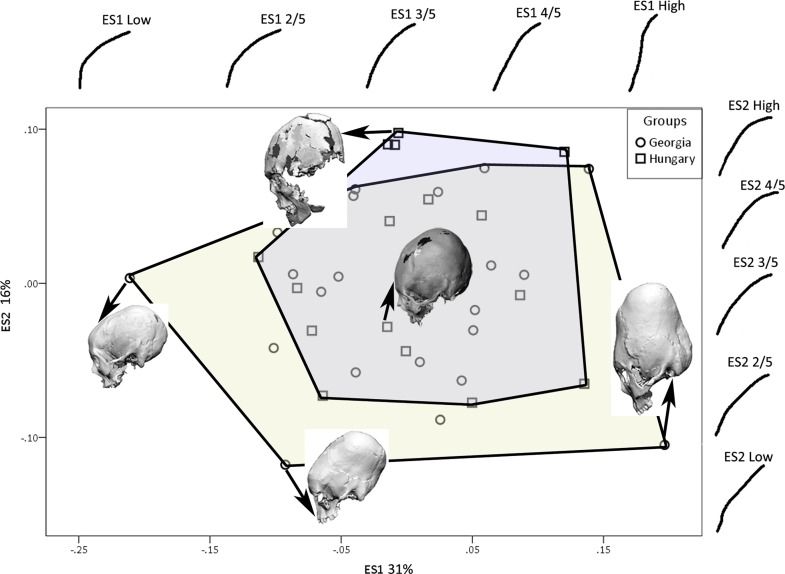
Eigenscores for the frontal outline plotted on ES1 and ES2 with convex hulls highlighting each group, also showing shape changes along the first and second morphospaces, and example crania demonstrating the variance.

**Fig 11:**
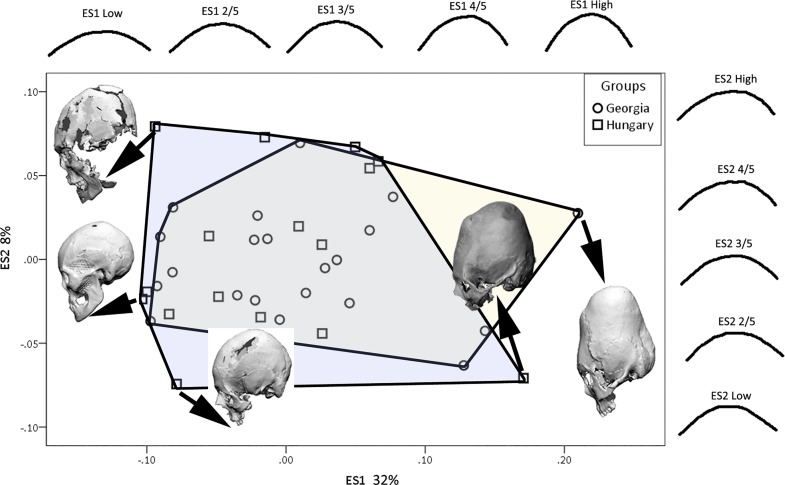
Eigenscores for the parietal outline plotted on ES1 and ES2 with convex hulls highlighting each group, also showing shape changes along the first and second morphospaces, and example crania demonstrating the variance.

**Fig 12:**
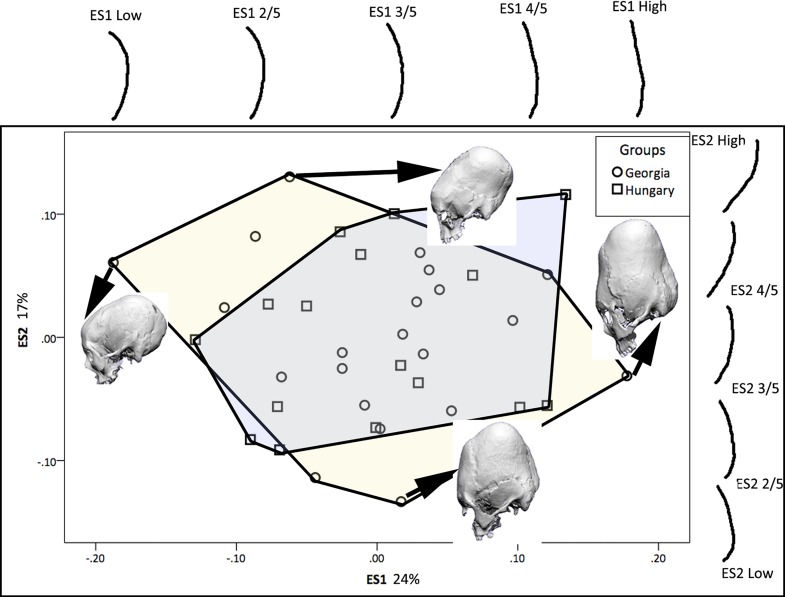
Eigenscores for the occipital outline plotted on ES1 and ES2 with convex hulls highlighting each group, also showing shape changes along the first and second morphospaces, and example crania demonstrating the variance.

In the second part of the analysis we compared the frontal, parietal and occipital outlines to study the shape differences in modified crania from Georgia and Hungary. In analysis of the frontal segment, demarcated by the glabella and bregma, the first two eigenshapes accounted for a total of 47% of variance (Fig 10). The first eigenshape (31%) charts the change from a low, rounded frontal to a tall, vertical frontal, while the second one marks the change from the frontal being angular at the anterior end to being angular at the posterior end. The parietal outline (Fig 11), going from bregma to lambda, picks up 32% of variance on the first eigenshape and shows the transformation from a low, shallow outline, to a tall, peaked outline. The second eigenshape with 8% of variance shows the change from a symmetrically outlined parietal to an obliquely angled and asymmetrically outlined parietal. The occipital outline, which in this study goes from lambda to the point on the occipital that forms a straight line from glabella to porion, covers 24% of variance on the first eigenshape (Fig 12) and charts the change from a curved to a vertically oriented occipital outline. The second eigenshape covering 17% of variance shows the transition from an anteriorly angled occipital outline on the negative end of the axis to a posteriorly angled outline on the positive end of the axis.

**Fig 13:**
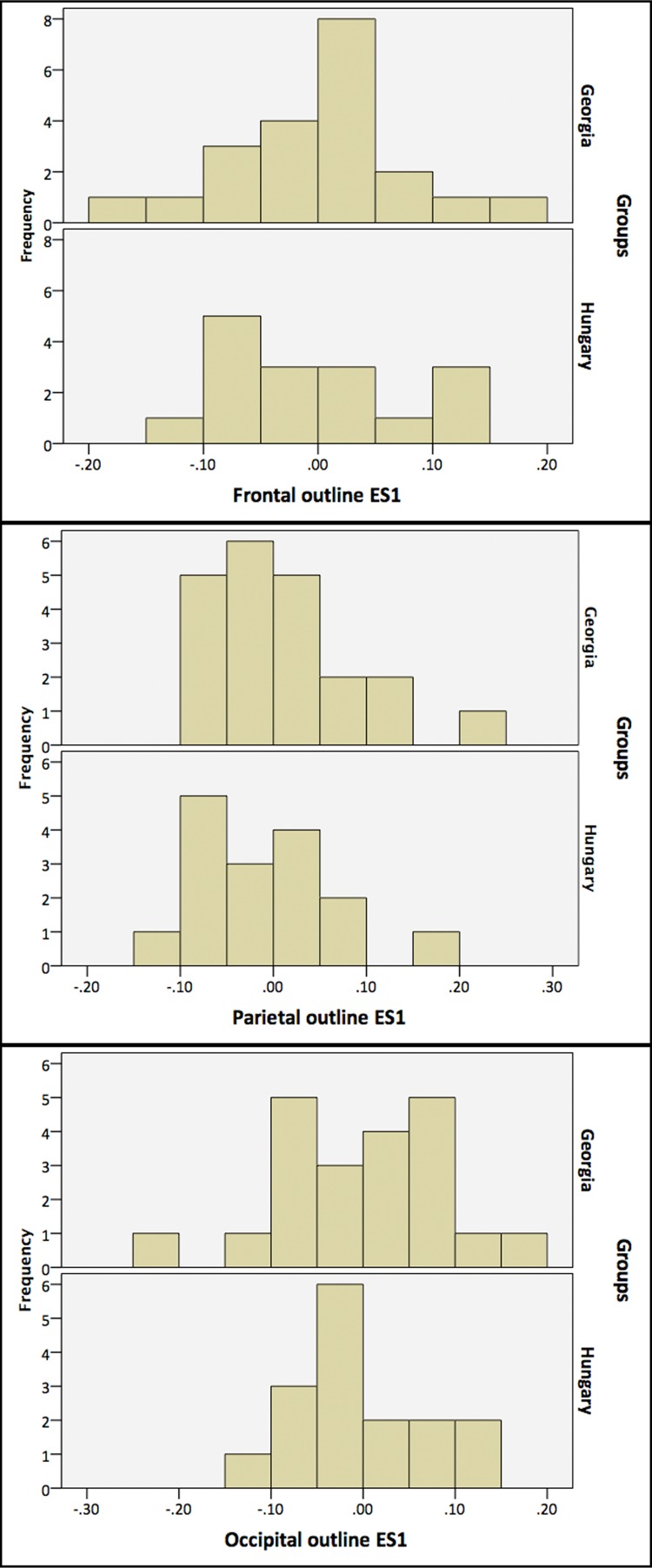
Histogram depicting the frequency of eigenscores on ES1 for the Georgian and Hungarian crania on frontal, parietal and occipital segments.

**Fig 14:**
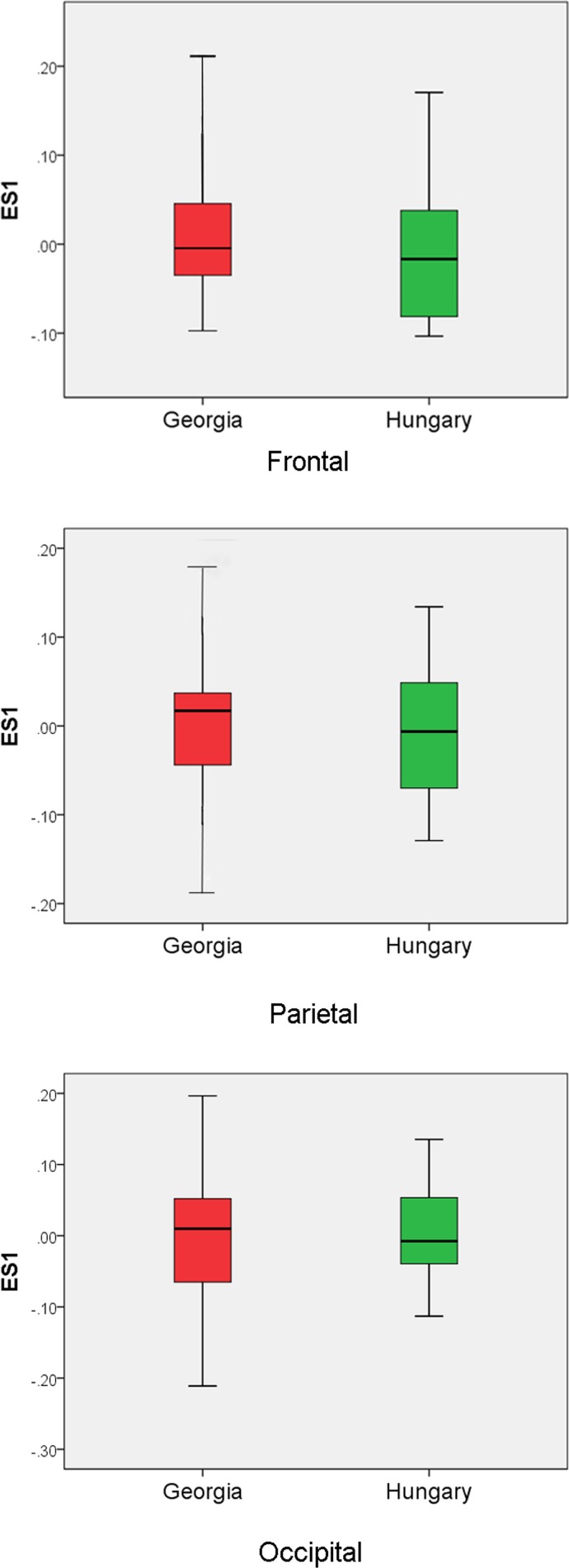
Box-and-whisker plots showing ES1 scores for the Georgian and Hungarian crania in frontal, parietal and occipital segments.

There is considerable overlap between the Georgian and Hungarian crania on plots of the first two eigenshapes (Figs 10–12). It is worth noting, however, that, on the whole, the Georgian crania lie at the outer edges of scatter. As can be seen from the example crania included in the plots, the Georgian crania encompass a wide range of variation in modified cranial shape. These include low and rounded frontal, parietal and occipital outlines, obliquely angled frontal and occipital with asymmetrical parietal outlines and much more vertically oriented frontal and occipital with tall, domed parietal outlines. The Hungarian modified crania fall within the range of cranial modification exhibited in Georgia, but in contrast with the Georgian crania they are constrained in showing gently sloping frontal, parietal and occipital outlines and do not show the extremes of low, high and oblique outline profiles seen in Georgia. The greater variance in the Georgian crania is also evident in the histograms and box-and-whisker plots depicting the scores of the crania on the eigenshapes. Fig 13 shows the frequency of scores for Georgian and Hungarian crania on ES1 for the frontal, parietal and occipital segments. Fig 14 shows the box-and-whisker plots for ES1. It is worth noting that in the region of the frontal and parietal the range around the mean is greater in Hungary even though the overall variance is greater in Georgia. Although not shown here, a similar pattern is repeated on all eigenshapes.

A discriminant function analysis using the eigenscores on all eigenshapes provides excellent and statistically significant separation of the Georgian and Hungarian crania (Table 3). On average the Georgian crania are classified at 78% accuracy and the Hungarian crania at 83% accuracy. Cross-validation does not reduce classification accuracy much. Georgian crania are still classified with an average of 78% accuracy and the Hungarian crania are classified with an average of 79% accuracy.

**Table 3:** Classification accuracy from discriminant analysis of frontal, parietal and occipital outlines.

	Georgia (N)	Hungary (N)	Percentage accuracy
**Frontal**			
Georgia	16	5	76%
Hungary	3	13	81%
**Cross validation**			
Georgia	16	5	76%
Hungary	3	13	81%
**Parietal**			
Georgia	16	5	76%
Hungary	2	14	88%
**Cross validation**			
Georgia	16	5	76%
Hungary	3	13	81%
**Occipital**			
Georgia	17	4	81%
Hungary	3	13	81%
**Cross validation**			
Georgia	17	4	81%
Hungary	4	12	75%

## Discussion

Intentional cranial modification as a cultural practice has been noted in Georgia since the Bronze Age, but after about 400 AD the practice became far more common. As this coincides with the start of the Migration Period and the ascendancy of the Huns with their signature modified heads in the Carpathian basin in Europe, our study sought to compare modified cranial shape in Georgia and Hungary to determine whether the Huns or other contemporaneous groups were responsible for this cultural practice in Georgia.

We found a distinct difference in the range of cranial shapes characterising Georgian and Hungarian crania. The Georgian crania showed greater variation in overall cranial outline and in the outline of individual cranial segments. The crania encompassed both shorter and taller varieties, ones that were highly compressed in the frontal and occipital regions and ones that were rounded and elongated from the frontal to occipital regions. In comparison, the Hungarian modified crania fell in the middle of the range of Georgian modified crania. They were characterised by relatively moderate frontal gradient, wide and shallow parietal outline and shallow occipital outline. This suggests that they were modified using the two bandage technique. This characteristic of Hungarian crania from the Migration Period has been recognized for some time. Pap [34–36] described the binding process at the site of Keszthely-Fenékpuszta as having one concentrically fitted bandage going from forehead to the nape of the neck across the temporal, and another bandage going from the crown of the head to continue under the chin. The second bandage caused a bregmatic depression around the coronal suture reducing cranial height while also reducing the height of the mandibular ramus and symphysis, and flattening the ramus. The greater variation in the range of curvature in the frontal and parietal segments as seen in the box-and-whisker plots in our study (Fig 14) suggests that the placement of the vertical bandage may have varied from prebregmatic to postbregmatic. Pap [36] suggested that the second bandage would not have hindered opening the mouth for food intake, but would have tightened the bandage to make it more effective.

It is significant that the two-bandage method restricting cranial height became widespread in the Transdanubian region between 4^th^– 7^th^ century AD following the period of Hunnic occupation, although multitudes of nomadic people in this region were engaged in the practice of intentional cranial modification prior to this time [2,13,15,20,31,58]. This attests to the strong influence of the Huns on the region and suggests that they promoted their identity by adopting a variation on an existing cultural custom. Where membership to the Hunnic community was inclusive without regard to genetic and ethnic background, this would suggest that the Huns were ingeniously able to differentiate themselves from other contemporary groups by standardising the technique of modification as a membership marker. Extensive literature on social identity theory suggests that diverse ethnic groups that are in close contact highlight differences by way of marking boundaries so as to strengthen their self-identity [68,69]. This could certainly apply to the Migration Period where several groups, described as nomads, barbarians or Germanic tribes, were in close proximity, yet often in conflict [70]. The heightened uptake of cranial modification in Migration Period Europe and variation in the style of modification could be interpreted as a way for the nomadic groups to define distinct social identities.

The skewed representation of predominantly adult females and the absence of juveniles with modified crania in the region of Mtskheta in Georgia suggests that the practice of cranial modification was not of local origin. This contrasts with the situation in Hungary where a complete demographic profile from juveniles to adults with intentionally modified crania exists. The situation in Mtskheta is similar to that in Germany, where Hakenbeck [15] suggested that the practice of female exogamy resulted in non-local females migrating from their place of birth and abandoning the custom of intentional cranial modification in their new community.

If the ubiquitous presence of modified crania with low cranial vault is considered to be a signifier of Hunnic identity, it may be surmised that Hunnic dominion and influence over Georgia from 4^th^– 7^th^ century AD was weak at best. The varied styles of annular modification encountered in the Mtskheta region suggest that there were multiple groups practising cranial modification during this time. Who then were these groups? It is difficult to answer this question with certainty but there is clear evidence that the Alans were present in the region of Mtskheta both prior to and during the time period under consideration. They are described in the Georgian historical chronicles as allies in strengthening the reign of Parnavaz and his son Saurmag (299–159 BC), in combating Armenian rulers, Artasan (72 AD) and Amazasp II (185–189 AD), in carrying out raids on the Georgian kingdom of Kartli during the reigns of Mirian (284–361 AD) and Vakhtang Gorgasali (447–552 AD), and in forming marital alliances with the Georgians [39]. The Alans had been practising cranial modification before the Huns arrived in Europe [18], which strengthens the possibility that some of the modified crania in Mtskheta belong to the Alans. The Georgian chronicles and classical historians such as Strabo and Pliny also describe the Sarmatians, who maintained strong connections with the Iberian kings of Georgia and helped them in their wars against Rome and Armenia [40]. These historical references, combined with deviations in the techniques of modification identified here, suggest that nomadic groups such as the Alans and Sarmatians in Mtskheta intensified the practice of intentional cranial modification as a way of differentiating themselves from the Huns and maintaining their social identity.

## Conclusion

The practice of intentional cranial modification was prevalent among the Indo-Iranian nomads such as the Scythians, Sarmatians, Alans and Gepids in the region of the Hungarian Plains, Eurasian Steppes and the Caucasus, prior to the arrival of Huns in Europe. After the arrival of Huns and with the start of the Migration Period the practice became far more common across Europe. Given the strong influence of the Huns on the events of the Migration Period, it is often assumed that modified skulls in Migration Period Europe signify the presence or the cultural influence of the Huns. Using extended eigenshape analysis in this study we found differences between the annular styles of modification utilized in Hungary and Georgia. The Hungarian modified crania were uniform in their pattern of modification and appear to have been modified using two bandages, which served to reduce cranial height. This strengthens our hypothesis that cranial shape was standardised in the Pontic steppe, which was the epicentre of Hunnic domination, so as to establish Hunnic identity. This was not the case in Georgia where modified crania in this study encompassed a range of variation from mild to extreme. This fits with the understanding that varied cultural influences distinct from those in Hungary operated simultaneously in Georgia. Our study finds little evidence for Huns or direct Hunnic influence in Georgia, but we suggest that existing nomadic groups such as the Alans and Sarmatians were responsible for the modified crania here. This conclusion also fits with the absence of textual references for Huns settling in Georgia, but can be accounted for by ample textual references for the presence of Alans and Sarmatians in the region. The Alans and Sarmatians were not immune to the influence of Huns, however. The Huns subjugated many, but not all, of them in the Hungarian plain. If there was any Hunnic influence in Georgia it was in the heightened uptake of the existing social custom of cranial modification in the Georgian nomadic groups.

## Supporting information

S1 File Georgia Hungary Modern.tps. Landmark data for each specimen.
(TPS)
